# Electromagnetic Navigational Bronchoscopy Learning Curve Regarding Pneumothorax Rate and Diagnostic Yield

**DOI:** 10.7759/cureus.58289

**Published:** 2024-04-15

**Authors:** Nicolas W Mwesigwa, Vasileios Tentzeris, Michael Gooseman, Suhail Qadri, Read Maxine, Michael Cowen

**Affiliations:** 1 Cardiothoracic Surgery, Hull University Teaching Hospitals, Hull, GBR; 2 Thoracic Surgery, Hull University Teaching Hospitals, Hull, GBR; 3 Thoracic Surgery, Castle Hill Hospital, Hull University Teaching Hospitals, Hull, GBR

**Keywords:** thoracic radiology, lung cancer screen, lesions of the lung parenchyma, electromagnetic navigation, ebus and interventional bronchoscopy

## Abstract

Electromagnetic navigational bronchoscopy (ENB) has emerged as an innovative technique for diagnosing peripheral and central nodules, offering an improved diagnostic yield compared to conventional bronchoscopy with fewer complications. That being said, pneumothorax remains a frequent complication. This retrospective study conducted at Castle Hill Hospital, UK, analysed ENB procedures over four years to assess the diagnostic yield and pneumothorax rates, exploring learning curves and procedural improvements specifically focusing on the diagnostic yield and pneumothorax rate as markers of change.

A total of 246 patients underwent 358 peripheral lung biopsies, revealing an overall diagnostic yield of 61.3%. The diagnostic yield increased from 58.2% in 2020-2021 to 66.0% in 2022-2023 while the pneumothorax rate decreased significantly from 9.8% to 3.4% (p = 0.021*). The majority of pneumothorax cases occurred following upper lobe procedures. The study depicts the importance of procedural experience in improving outcomes, suggesting a learning curve effect. Additionally, it emphasizes the potential for technological advancements, such as robotic assistance, to mitigate operator-dependent variability and improve reproducibility in ENB procedures. These findings contribute to optimizing diagnostic pathways for lung lesions and improving patient safety in ENB interventions.

## Introduction

Electromagnetic navigational bronchoscopy (ENB) stands out as one of the most advanced bronchoscopy techniques available. As delineated by Pickering and colleagues, ENB is characterised by a pre-procedural chest CT of the patient that creates a three-dimensional (3D) virtual airway map through which the surgeon navigates to locate lung nodules for biopsy as well as interventions such as dye marking before resection [1]. It has a notably superior diagnostic yield compared to conventional bronchoscopy; however, the occurrence of pneumothorax remains frequent with variability in diagnostic yield [2,3]. Our study explored the diagnostic yield and pneumothorax rate of ENB at Castle Hill Hospital, UK, exploring learning curves and changes in statistics over four years. As demonstrated by Lee et al., surgical skills drastically improve with increased frequency of performing a procedure. This article exhibits a clear procedure learning curve that simultaneously improved the ENB diagnostic yield and reduced the pneumothorax rate at our hospital.

## Materials and methods

Electromagnetic navigational bronchoscopy (ENB) has become an important component of our facility's lung cancer pathway, routinely performed following referrals from our weekly Lung Cancer Multidisciplinary Team (MDT) meetings. These sessions usually involved respiratory physicians, radiologists, thoracic surgeons, oncologists, as well as clinical nurse specialists. Patient eligibility for ENB is carefully evaluated, with considerations including the presence of a bronchus leading to the lesion (positive bronchus sign), lesion depth, size, and suitability for alternative diagnostic procedures such as CT-guided biopsy or diagnostic wedge resection.

In this retrospective analysis, we analysed the ENB outcomes of 246 patients who underwent 358 peripheral lung biopsies between January 2020 and September 2023. This indicates that multiple patients had more than one lobe or lesion biopsied in the same sitting, therefore, for the occurrence of pneumothorax, each lesion biopsied was considered separately. The distribution of biopsies indicated 108 sampled from January 2020 to December 2021, and 250 from January 2022 to September 2023.

To assess the diagnostic efficacy of ENB, we scrutinized true-positive, true-negative, false-positive, and false-negative results, stratifying our analysis for the two distinct periods (2020-2021 and 2022-2023), to assess changes following the performance of a considerable number of procedures. Regarding pneumothoraxes following the procedures, we assessed all post-procedural chest X-rays performed with attached radiology reports to ascertain their occurrence. For accuracy, for patients who had more than one pneumothorax following a biopsy of different lobes (left and right), we recorded these as two. Patients were unable to have more than two pneumothoraxes recorded per procedure even following more than two lesions biopsied with bilateral pneumothoraxes. For this study, we did not explore the percentage/number of chest drain insertions following pneumothorax occurrence.

This thorough retrospective examination aims to provide nuanced insights into learning curves associated with this procedure. By discerning trends in diagnostic yield and pneumothorax incidence across different periods, we endeavour to identify potential areas for procedural enhancement and optimization. The findings derived from this analysis will serve as the basis for future research into aspects that could make this procedure more successful with fewer complications.

## Results

Approximately 32.5% (80/246) of the biopsies were cancerous lesions (Table 1). This excluded results that depicted atypical cells (*), most of which proceeded to show cancer following definitive surgery.

**Table 1:** Patient characteristics and lobar distribution of lesions Values are presented as mean ± SD or counts, (%) unless otherwise indicated. UL-upper lobe; LL-lower lobe; RML-right middle lobe *Results depicting atypical cells were excluded from analysis; however, the majority of these proved to be cancerous lesions.

Variables	Total patients, n	All lobes	UL	LL	RML	p-value
Baseline characteristics						
Age, mean ± SD	246	68.07 ± 9.23	68.07 ± 9.23	67.98 ± 9.32	67.26 ± 9.35	0.458
Size (cm), mean ± SD	246	1.13 ± 1.34	1.13 ± 1.34	1.14 ± 1.35	1.08 ± 0.57	0.219
Cancerous lesions, n (%)*	246	80 (32.5)	62 (77.5)	12 (15.0)	6 (7.5)	0.053
Pneumothorax rate	358	24 (6.7)	18 (75.0)	3 (12.5)	3 (12.5)	0.198

The mean size of the lesion biopsied was 1.13 cm, much smaller than previous studies. The overall diagnostic yield was 61.3% with a positive predictive value of 98.7% (Tables 2, 3).

**Table 2:** Relevant definitions

True positives	Navigational bronchoscopy detected specific tumours (Adeno, SC, carcinoid, lymphoma…), which was further confirmed on a surgical procedure.
True Negatives	Navigational bronchoscopy suggested no malignancy or inflammatory lesion. This was confirmed with CT follow-up, which showed a decrease in the size of the lesion till discharge or wedge resection, which indicated no malignancy. It also included inflammatory/infective lesions successfully treated on follow-up.
False Positives	Navigational bronchoscopy suggested a malignant lesion, which was negative on definitive surgery.
False Negatives	Navigational bronchoscopy suggested no malignancy/inconclusive/inflammatory lesions; however, subsequent histology proved malignancy.

**Table 3:** Overall diagnostic yield and positive predictive value

Overall		
True positive = 80	True negative = 71	True diagnostic (TN+TP) = 151 (TD)
False positive = 1	False negative = 94	Non-diagnostic (FP+FN) = 95 (ND)
Overall diagnostic yield	TD/(TD+ND) X 100	61.3%
Positive predictive value	TP/(TP+FP) X 100	98.7%

In the 2020-2021 period, the diagnostic yield was 58.2%, which improved to 66.0% in the 2022-2023 period (Tables 4, 5).

**Table 4:** Diagnostic yield and positive predictive value for the period of 2020 to 2021

2020-2021		
True positive = 55	True negative = 30	True diagnostic (TN+TP) = 85 (TD)
False positive = 1	False negative = 60	Non-diagnostic (FP+FN) = 61 (ND)
Diagnostic yield (2020-2021)	TD/(TD+ND) X100	58.2%
Positive predictive value	TP/(TP+FP) X 100	98.2%

**Table 5:** Diagnostic yield and positive predictive value for the period of 2022 to 2023

2022-2023		
True positive = 25	True negative = 41	True diagnostic (TN+TP) = 66 (TD)
False positive = 0	False negative = 34	Non-diagnostic (FP+FN) = 34 (ND)
Diagnostic yield (2022-2023)	TD/(TD+ND) X100	66%
Positive predictive value	TP/(TP+FP) X100	100%

**Figure 1:**
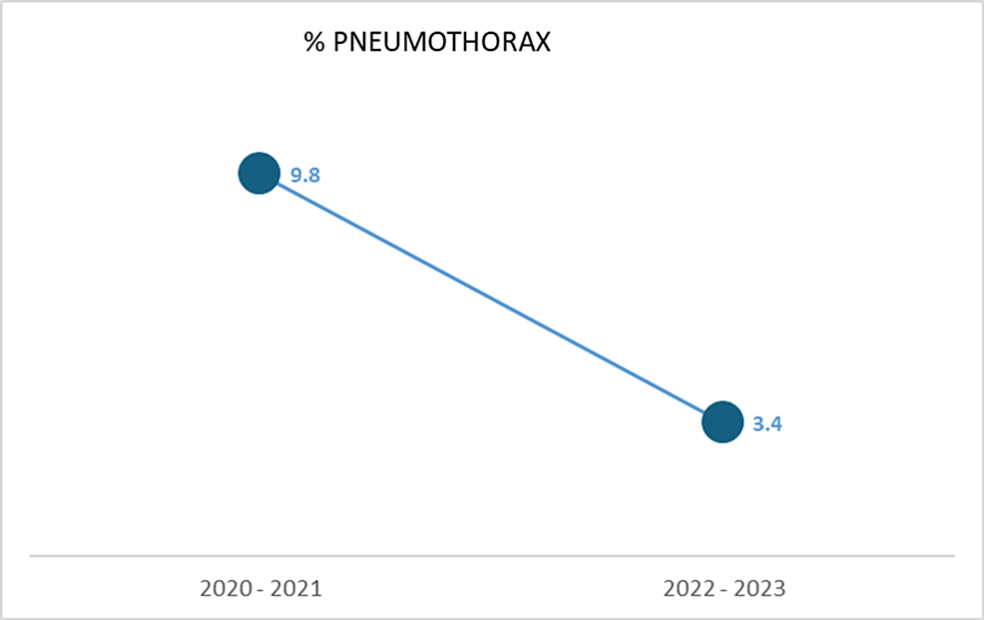
Graph depicting the change in pneumothorax rate over time

The pneumothorax rate meanwhile was 9.8% in the 2020-2021 period and drastically reduced to 3.4% in the 2022-2023 period (p = 0.021*) (Table 6 and Figure 1). The majority of these occur in the upper lobe regions. The overall pneumothorax rate averaged 6.7% mainly attributed to the initial high rate of occurrence.

**Table 6:** Pneumothorax rate by the period of the ENB procedure ENB: electromagnetic navigational bronchoscopy

Period	Pneumothorax rate (%)	p-value
2020-2021	9.8	0.021*
2022-2023	3.4	

These results show a general reduction in pneumothorax occurrence with an associated improvement in diagnostic yield.

## Discussion

Lung cancer has been attributed to be the leading cause of cancer-related deaths worldwide with poor prognosis mainly due to late diagnosis. It has been conferred an overall five-year survival after diagnosis to be 17% [4]. Greater use of routine chest CT scans as well as the rollout of national lung cancer screening programs in the United Kingdom and other regions of the world has led to increased detection of lung nodules. As most lung lesions visualized on routine scans in patients of low-risk profiles are benign, there is a high demand for minimally invasive techniques to accurately diagnose these lesions with minimal complications conferred to the patient [5]. These technologies include but are not limited to CT-guided biopsy, fluoroscopic-assisted bronchoscopy, endobronchial ultrasound, navigational bronchoscopy, and, much more recently, robotic-assisted bronchoscopy.

Percutaneous computerized tomography-guided lung biopsy has been the mainstay regarding minimally invasive lung biopsy techniques employed historically, however, not without complications. The technique of passing a needle through the rib spaces to obtain fragments of diseased lung tissue has been reported as early as 1940 (Craver) as well as Craver and Binkley (1939). Following reports of early success, there were a series of reported fatalities during the 1960s and 1970s attributed to pneumothoraxes and haemorrhage [6]. There has been significant improvement in this technique with CT-guided biopsies first reported by Haaga and Alfidi (1976) with improving accuracy, sensitivity and specificity in detecting primary lung cancer [6]. Despite this, a high pneumothorax rate has persisted with this technique with some meta-analyses placing this at 25.9% and chest drain insertion at 6.9% [7]. These studies identified major factors altering pneumothorax rate such as patient positioning, when planning and performing CT-guided biopsy.

Parallel to this, bronchoscopy techniques have been used and gradually evolved from diagnosis of central airway lung lesions to further bronchial lesions to peripheral and central lung parenchymal lesions. Initially, the main indications for bronchoscopy included removal of foreign bodies and dilation of strictures from tuberculosis and diphtheria [8]. Following the initial use of rigid bronchoscopy in the early part of the twentieth century, fibreoptic bronchoscopy (FOB) was developed in the late 1960s becoming the mainstay for diagnosis of different airway tumour types [9]. The development of video bronchoscopes aided teaching and demonstration of airway lesions making it a highly interactive procedure for trainees. The flexibility of the scope also allowed for inspection of smaller airways up to the sixth order improving the visualisation and characterisation of lesions [8]. Important to note that FOB has been mainly employed in the diagnosis of airway lesions. Sampling of peripheral lung lesions depends on several factors including lesion size, distance from the hilum and the relationship between the lesion and the bronchus [8]. Diagnostic yields vary depending on these factors with studies depicting lower yields for lesions <3 cm from 14-50%. It is also quite clear that the positive bronchus sign predicts a much higher yield for bronchoscopy biopsy of peripheral lung lesions. It has been shown that fluoroscopy increases the yield of conventional bronchial biopsy procedures, however, it remains time-consuming and not widely available [8].

Electromagnetic navigational bronchoscopy (ENB) is a novel bronchoscopy technique that utilizes 3D mapping software to delineate pathways used by operators to navigate toward specific lesions in the lung parenchyma for both diagnostic and therapeutic purposes [10]. The system employs a localisation device that helps place bronchoscopy accessories in target locations. It is a relatively recent advancement with a few centres in the United Kingdom currently employing this technique. It confers an advantage to conventional CT-guided biopsy, as it allows operators to navigate to both peripheral and central lung lesions with less risk of complications, mainly pneumothorax. It also provides superiority to FOB due to higher diagnostic yield conferred to even smaller lesions. The procedure was initially confirmed safe with only an additional operational procedural time of 15 minutes on average as compared to conventional bronchoscopy [11]. As compared to conventional bronchoscopy, it was also seen that ENB produced a higher diagnostic yield of about 74% for peripheral lung lesions and 100% for lymph nodes. Notably, this was independent of the size of the lesion [12]. Most of the initial studies on ENB were performed under fluoroscopy to theoretically improve the diagnostic yield of these procedures, as it showed for conventional bronchoscopy techniques. It is now known that standalone ENB can be performed without compromising diagnostic yield or increasing complication risk [13]. Further studies have also confirmed the redundancy of fluoroscopy in ENB procedures, which would confer additional time to the procedure without the additional benefit of improved diagnostic yield [14]. It is important to note that it has been shown endobronchial ultrasound (EBUS)-guided transbronchial biopsy has been shown to be superior to conventional diagnostic techniques and can contribute to a reduction in patient discomfort with improved accuracy of diagnosis [15].

In our study, we sought to investigate the rate of occurrence of pneumothoraxes, mainly looking at its change over time to ascertain whether this technology should be continually justified in diagnostic pathways of lung lesions. There is still a wide discrepancy in the rate of pneumothorax following navigational bronchoscopy with some studies noting up to 10% rate compared to others putting at about 1-3%. This relatively low rate in comparison to CT-guided biopsy techniques is mainly attributed to the avoidance of pleural breach during ENB [10].

We also sought to study the change in diagnostic yield over the same period as the current published diagnostic yield is also a variable, ranging from 59% to 77.3% [10].

## Conclusions

This study highlights the smooth surgical learning curve involved in conducting ENB procedures. The more procedures the surgeon conducts, the better the outcomes in terms of both diagnostic yield and pneumothorax rates. Following deeper analysis, factors contributing to this change include a better selection of patients with positive bronchus signs, an improved planning stage with accurate targeting of lesions and a selection of simpler airways. Ensuring screening CT scans are performed with protocols necessary for ENB software has reduced the number of scans required with earlier decision-making on whether said patients are suitable for ENB. Most certainly, improved surgical technique and familiarity have been a major factor in these improved statistics associated with improved understanding and interpretation of scans by our radiology colleagues to better identify suitable candidates for this procedure.

Our current diagnostic yield of 66.0% and pneumothorax rate of 3.4% were comparable to previous studies. While the ENB procedure continues to improve at our hospital, over-dependence on the surgical skills of the operator still creates greater operator variability, which is a limitation. This could be solved by introducing robotic technologies with less operator dependence that improve the reproducibility of the procedure across different operators/surgeons.
